# Prevalence and associated factors of inappropriate hospital admissions and days of children in a secondary hospital in Shanghai, China

**DOI:** 10.1371/journal.pone.0275645

**Published:** 2022-10-06

**Authors:** Wenwei Liu, Huimin Zhang, Haichen Zhang, Tongzhou Lyu, Suwei Yuan

**Affiliations:** 1 College of Philosophy, Law and Political Science, Shanghai Normal University, Shanghai, China; 2 School of International and Public Affairs, Shanghai Jiao Tong University, Shanghai, China; 3 Shanghai Xuhui Mental Health Center, Shanghai, China; 4 School of Politics and International Relations, East China Normal University, Shanghai, China; 5 Office of the Director, Ren Ji Hospital, Shanghai Jiao Tong University School of Medicine, Shanghai, China; 6 China Hospital Development Institute, Shanghai Jiao Tong University, Shanghai, China; UAB Hospital, UNITED STATES

## Abstract

**Background:**

Although the appropriateness of hospital utilization of adults and the elderly in China was audited by several studies, the appropriateness of hospital use by children in Shanghai remains to be determined. This study aims to assess the level of inappropriate hospital admissions and hospital days, to detect factors associated with inappropriateness, and to elucidate reasons for inappropriateness.

**Methods:**

A retrospective review of the records of 291 admissions and 1449 hospital days of children inpatients from a secondary hospital in Shanghai was performed by two reviewers using the Chinese version Pediatric Appropriateness Evaluation Protocol (C-PAEP). Demographics, socio-economic characteristics, and other admission- or hospital stay-related information were collected and analyzed to determine factors associated with inappropriateness utilizing multivariate regression models.

**Results:**

38.5% (n = 112) of admissions and 9.5% (n = 137) of hospital days were categorized as inappropriate, according to the C-PAEP. Children who were non-Shanghai residents (*p* < 0.001), admitted through the emergency sector (*p* = 0.030), and/or received services in a surgical ward (*p* < 0.001) had a higher risk of being admitted inappropriately. Payment method (*p* = 0.006), service type (*p* < 0.001), comorbidity (*p* = 0.016), length of stay (*p* = 0.007), and appropriateness of admission (*p* < 0.001) were found to be associated with prevalence of inappropriate hospital days. Approximately three-fourths of the inappropriate admissions were premature admissions (75.9%, n = 85). The most frequent reasons for inappropriate hospital days were awaiting test results (34.3%, n = 47), awaiting surgery (19.7%, n = 27), awaiting test execution (10.9%, n = 15), and family unprepared for home care (10.9%, n = 15).

**Conclusions:**

Although the extent of inappropriate hospital days was moderate compared with that found by previous investigations, the prevalence of inappropriateness of admission was considerable. To enhance the appropriateness of hospital care for children, interventions could be implemented according to the associated factors and identified causes.

## Background

Hospitals are the main health care service providers in China. In 2018, hospital expenditures were 4000.7 billion CNY, which constitutes 77.6% of the overall expenditures of health institutions in China [1]. It is believed that inpatient care is one of the driving forces of rapidly growing health expenses. From 2016 to 2018, the number of hospital admissions increased by 14.2% in China, and the cost of each admission rose by 8.4% [1, 2]. Containing dramatically increasing hospital costs, especially hospital inpatient costs, constitutes one of the core objectives of China’s health system reforms [3].

To alleviate the burden on health costs without compromising quality of care, one effective approach is to target inappropriate use of hospital care. To achieve this, numerous measures and methods have been developed. One such method is the utilization review (UR). UR projects use standard instruments to assess the necessity or appropriateness of utilizing specific health service resources [4]. One of the most employed UR instruments is the Appropriateness Evaluation Protocol (AEP). The original AEP was developed by Gertman and Restuccia in the United States for screening acute adult hospitalizations [5].

Realizing that different criteria might be necessary for pediatric practice, Kreger and Restuccia developed the Pediatric AEP (PAEP) on the basis of the adult AEP [6]. The original PAEP comprises two major parts, admission criteria and hospital days criteria. There are 21 and 28 descriptive criteria, each of which justifies an appropriate admission or hospital day, respectively. When an admission or a hospitalization day fails to satisfy all of the criteria, it can be categorized as inappropriate. Conversely, if an admission or a hospital day satisfies at least one criterion, it can be audited as appropriate. Usually, two assessors are required for UR. PAEP has been adapted globally to assess the appropriateness of hospitalizations. The level of inappropriate admissions and hospital days varies from 0% to 40.7% [6–13], and from 14.3% to 55.5% [8, 11, 12, 14, 15], respectively, depending on several factors. The primary determinants of inappropriateness identified in these studies include age, service type, admission route, etc. However, few investigations have reported the appropriateness of hospital utilization in developing countries [9, 16, 17], and no pediatric hospital utilization review project has yet been carried out in China.

To contain rapidly-growing hospital expenditures, in Shanghai, instead of targeting inappropriate hospital use, the basic medical insurance fund management department negotiates with hospitals via global budgeting. The budget is usually calculated on the basis of previous insurance fund usage. When the cost exceeds the budget, the provider is mandated to share a certain percentage with the insurance [inappropriate hospital days of a tertiary hospital in Shanghai, China]. Consequently, there is a possibility that inappropriate use can emerge when the containment measure aims to limit the expenditure as a whole. In 2018, children (aged under 14) constituted a considerable 14.0% of discharged inpatients from hospitals [1]. Although the appropriateness of hospital utilization of adults and the elderly in China was audited by several studies [18–21], the appropriateness of hospital use by children remains undetermined. This study aims to provide the first empirical evidence of children’s inappropriate hospital use in Shanghai, China. The objectives of this study are as follows: (1) to determine the level of inappropriate hospital admissions and hospital days of children in a secondary hospital in Shanghai; (2) to detect factors associated with inappropriateness; and (3) to elucidate reasons for inappropriateness.

## Materials and methods

### Study design

A secondary hospital, located in central Shanghai, agreed to participate in this study. This hospital is a university-affiliated secondary-teaching hospital comprising 18 clinical departments and 1036 beds. All departments agreed to participate in this review project. The records of all children patients (n = 334) admitted from 1 June 2016 to 1 June 2019 were extracted from the electronic patient record system. The review process started on 5 August 2019 and ended on 30 October 2019. The study was exempted from ethical review by the China Hospital Development Institute, Shanghai Jiao Tong University. The two hospitals are teaching hospitals (affiliated with Shanghai Jiao Tong University) that provide both care services and health related education. Once a patient is admitted to the hospitals, the agreement of providing his or her personal and medical information for academic use is established by mutual consent. We also signed a contract with the Academic Ethics Board of each hospital to guarantee that the data shall be used only for academic purposes. In addition, we obtained oral consent from all of the children’s guardians, and the medical records were anonymized and de-identified through the review and analysis procedure.

**Fig 1:**
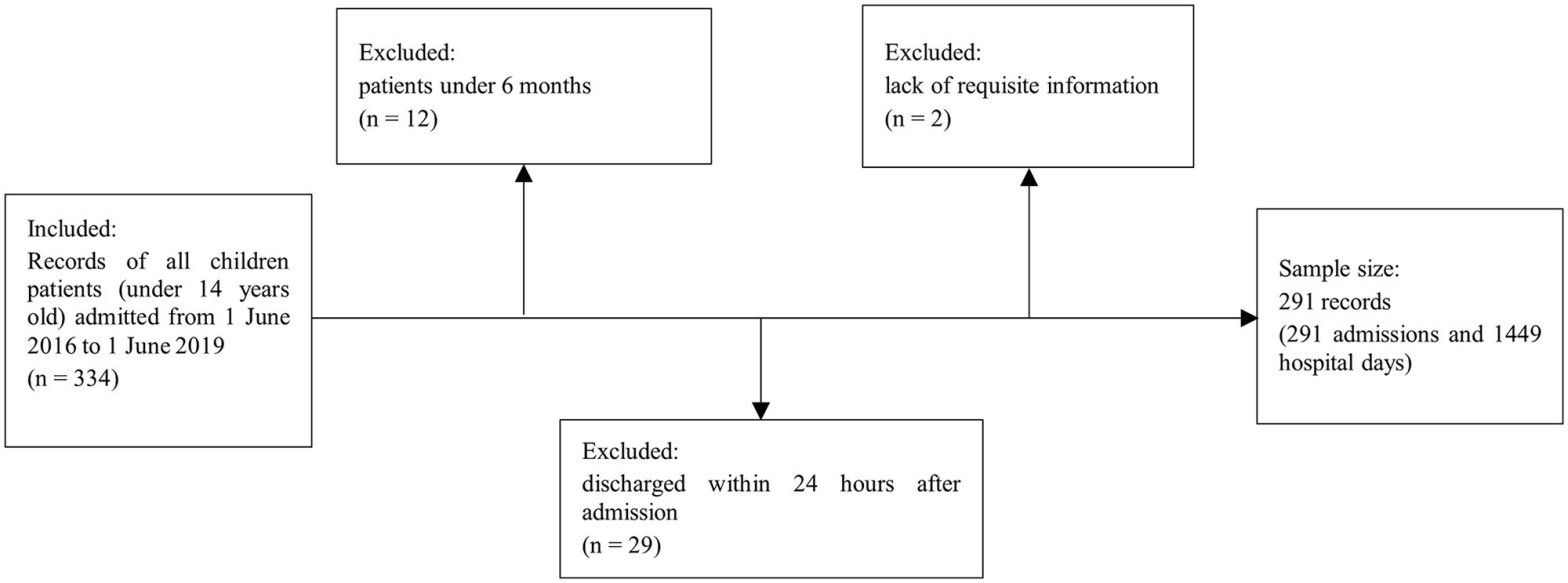
Identification for inclusion and exclusion of records.

The original PAEP was developed to assess the appropriateness of inpatients from 6 months- to 15 years-old [6]. However, according to China’s medical practice, only patients from 6 months- to 14 years-old can be admitted as pediatric inpatients. Finally, 12 records of patients aged under 6 months, 29 records of patients discharged within 24 hours after admission, and 2 records that lacked requisite information for assessment were excluded (Fig 1).

### The Chinese version PAEP

Studies have demonstrated that direct use of the PAEP without adaptation to specific contexts of practice can result in low validity [22]. Considering differences in health systems and medical practice, adaption of the original PAEP to the specific Chinese specific context was necessary.

By following the adaptation process of the original AEP to the Chinese Version AEP (C-AEP) [23], the original PAEP was chosen for adaptation. The first step involved forward and backward translations conducted by two bilingual Ph.D. students of clinical medicine. Each difference that occurred in translation was discussed until a consensus was reached. The translated Chinese version PAEP was then sent to a pediatric expert panel comprised of 20 pediatricians with 10 or more years of working experience. By consensus of the panel, each item of the Chinese version of the original PAEP was assessed to determine if it would remain the same, be modified, or be deleted.

Only small modifications were made to the original PAEP. One item each was deleted from the admission and hospital day part, respectively, due to a “lack of severity”, according to the expert panel. These criteria are “intravenous medication of antibiotics at least every 8 hours” and “intravenous medication and/or subcutaneous injections at least twice daily”. Two criteria from the admission part were modified according to China’s pediatric practice. Specifically, the item “persistent fever for more than 10 days” was modified to “persistent fever for more than 5 days”. In addition, “severe sepsis” and “acute abdomen” were added to the conditions (not responding to outpatient management) that should be admitted. Ultimately, the Chinese version PAEP (C-PAEP) contained two parts: admission criteria and hospital days criteria. Consequently, there are 47 objective criteria (20 and 27 for admissions and hospital days, respectively) in the C-PAEP (see S1 File).

Reliability and validity were tested in a relatively small sample size of randomly selected 50 admissions and 50 days of care in the pilot study. The overall agreements of admissions and hospital days by the two assessors were 78.0% and 90.0%, respectively. The specific agreements of appropriateness of admissions and hospital days were 62.1% and 70.6%, respectively. Cohen’s Kappa statistic for the C-AEP reviewers was 0.563 (95% confidence interval [CI] = 0.281–0.845) and 0.760 (95% CI = 0.566–0.954) for the admission and hospital stay part, respectively. To test the convergent validity of the C-PAEP, two pediatricians from another hospital were invited to assess appropriateness according to their own professional knowledge and experience. The results of the assessments made by the physicians and the reviewers were compared. The overall and specific agreements of the judgments by the physicians and reviewers were 88.0% and 71.9% for admissions and 82.0% and 52.9% for hospital days, respectively. The Kappa statistic for the two groups of reviewers were 0.641 (95% CI = 0.429–0.853) and 0.562 (95% CI = 0.321–0.803) for the admission and the hospital stay part, respectively. The sensitivity and specificity of the criteria were 79.3% and 87.5% for the admission part, and 75% and 84.2% for the hospital days part, respectively. In general, the C-PAEP constitutes a sensitive and specific instrument of adequate reliability and validity for elucidating the appropriateness of hospital use by children.

An admission or a hospital day is categorized as appropriate if any of the objective items are met. However, if an admission or a day of stay fails to satisfy any of the items, the admission or the hospital day will be identified as inappropriate hospital use. The judgements of appropriateness of admission and hospital stay are independent. Specifically, when an admission is classified as inappropriate, the hospital days can still be appropriate, and vice versa. For example, when a patient is admitted appropriately, even if he or she has to wait for test results without any service or condition conforming to the criteria of the C-PAEP, the hospital days could be judged as inappropriate. In addition to the objective criteria, the AEP also includes a subjective override option, which allows the auditor to use personal knowledge and/or professional experience to override the objective criteria. The average time for assessing a single record (including the admission and the whole stay) was approximately 10 to 20 minutes, depending on the length of the stay and the complexity of the case. The two reviewers involved in the pilot study concerning reliability and validity were invited to participate in the review project. Both assessors possess previous experience in auditing medical records using the Chinese version AEP (C-AEP). The reviewers were required to audit the records independently. At the end of each day, a meeting was held for the reviewers, and the results were compared and discussed until a consensus was reached. The developers of PAEP stated that the reasons for inappropriateness of children hospitalizations were similar to those of adult hospitalizations [6]. Therefore, the C-PAEP assessors were also required to write down the reason for inappropriateness, according to the C-AEP reason lists for adults [23].

### Data collection

Demographic and socio-economic characteristics, including gender, age, residence, and payment method, were collected. Age was recoded into a tripartite categorical (6 months- to 5 years-old, 5 to 9.5 years-old, and 9.5 to 14 years-old) variable because former studies have shown more sensitivity to inappropriateness [9]. Residence refers to the residential status of the child, i.e., whether or not the child is a resident of Shanghai. Admission- and hospitalization-related factors, including day of admission, admission time, readmission, service type, comorbidity, and length of stay were also collected by the reviewers. Day of admission was categorized into holidays and working days, according to the national holiday announcements made by the State Council of the People’s Republic of China. Admission time was divided into three periods, according to working shift regulations of the hospital (8:00 to 11:59, 12:00 to 17:59, and 18:00 to 7:59).

### Statistical analysis

Percentages were employed in the descriptive analysis for categorical variables, and average and standard deviation, as well as median and interquartile range (IQR) where necessary, were employed for continuous variables. Missing values were reported in the descriptive table. The significance of differences was tested by Chi-square test, Student’s *t*-test, one-way analysis of variance, and Pearson’s correlation test in univariate analysis, accordingly. Variables that exhibited a significant association with inappropriateness in the univariate study were introduced to the multiple regression analysis. The stepwise logistic regression model and the ordinary least-squares regression model were utilized to explore factors associated with inappropriate admission and prevalence of inappropriate hospital days in multivariate analysis, respectively. Goodness-of-fit was tested and measured by the Hosmer and Lemeshow test and adjusted R-squared statistic, respectively. A *p*-value < 0.05 was considered statistically significant. Microsoft Office Excel version 2010 and SPSS version 20.0 were used for data entry and analysis, respectively.

## Results

### Inappropriate admissions and hospital days

A total of 291 records were included in the review. 53.6% (n = 156) of the patients were boys. The average age was 11.1 (± 2.816). Younger age (6 months- to 5 years-old), median age (5 to 9.5 years-old), and older age (9.5 to 14 years-old) children comprised 4.8% (n = 14), 24.1% (n = 70), and 71.1% (n = 207), respectively. Approximately half (51.2%, n = 149) of the patients were Shanghai residents, and 62.5% (n = 182) were self-pay patients. 30.2% (n = 88) of the children were admitted on holidays. 62.6% (n = 181) of the admissions occurred from 08:00–11:59, 34.9% (n = 101) from 12:00–17:59, and 2.4% (n = 7) from 18:00–07:59. Readmissions counted for 40.2% (n = 117). 30.2% (n = 88) of the children were with at least one comorbidity, and 50.2% (n = 146) received surgical services. The average and median length of stay was 4.98 days (± 6.58) and 3.00 days (IQR = 4.00), respectively.

Among the 291 records and the consecutive 1449 days of care reviewed, 112 (38.5%) admissions and 137 (9.5%) hospital days were categorized as inappropriate, according to the C-PAEP. The distribution of inappropriate admissions and hospital days by inpatients’ characteristics is presented in Table 1. The distribution of inappropriate admissions in different gender, age, day of admission, admission time, comorbidity, and payment method groups were not significant. Inpatients who lived in Shanghai were admitted more appropriately than were non-Shanghai children (*p* < 0.001). Patients admitted through the outpatient sector had a significantly lower proportion of being admitted inappropriately (*p* = 0.035). Regarding the distribution of inappropriate hospital days, female (*p* < 0.001), self-pay (*p* < 0.001), admitted through the emergency sector (*p* < 0.001), and readmitted (*p* < 0.001) children were reported to have significantly more inappropriate hospital days. The average lengths of appropriate and inappropriate hospital days were 4.51 (± 0.33) and 3.04 (± 3.78), respectively.

**Table 1:** Distribution of inappropriate admissions and hospital days by patients’ characteristics.

	Admissions			Hospital stay		
	Appropriate n (%)	Inappropriate n (%)	*p*	Appropriate n (%)	Inappropriate n (%)	*p*
Gender						
Male	98 (62.8)	58 (37.2)	0.622	583 (93.9)	38 (6.1)	<0.001
Female	81 (60.0)	54 (40.0)		729 (88.0)	99 (12.0)	
Age						
0.5 < and ≤ 5	10 (71.4)	4 (28.6)	0.198	19 (95.0)	1 (5.0)	0.786
5 < and ≤ 9.5	37 (52.9)	33 (47.1)		212 (95.1)	11 (4.9)	
9.5 < and ≤ 14	132 (63.8)	75 (36.2)		1081 (89.6)	125 (10.4)	
Residence						
Shanghai	108 (72.5)	41 (27.5)	<0.001	647 (89.7)	74 (10.3)	0.347
Non-Shanghai	71 (50.0)	71 (50.0)		665 (91.3)	63 (8.7)	
Payment method						
Self-pay	109 (59.9)	73 (40.1)	0.462	676 (87.3)	98 (12.7)	<0.001
Insured	70 (64.2)	39 (35.8)		636 (94.2)	39 (5.8)	
Day of admission						
Holiday	59 (67.0)	29 (33.0)	0.201	-	-	
Working day	120 (59.1)	83 (40.9)				
Admission time	(missing = 2)					
08:00–11:59	113 (62.4)	68 (37.8)	0.287	-	-	
12:00–17:59	58 (47.4)	43 (42.8)				
18:00–07:59	6 (85.7)	1 (14.3)				
Admission route						
Outpatient	168 (63.4)	97 (36.6)	0.035	1032 (90.9)	103 (9.1)	<0.001
Emergency	11 (42.3)	15 (57.7)		280 (89.2)	34 (10.8)	
Readmission						
Yes	81 (69.2)	36 (30.8)	0.026	714 (88.7)	91 (11.3)	<0.001
No	98 (56.3)	76 (43.7)		598 (92.9)	46 (7.1)	
Service type						
Surgical	67 (45.9)	79 (54.1)	<0.001	496 (95.2)	25 (4.8)	<0.001
Medical	112 (77.2)	33 (22.8)		816 (87.9)	112 (12.1)	
Comorbidity						
Yes	58 (65.9)	30 (34.1)	0.310	570 (96.4)	21 (3.6)	<0.001
No	121 (59.6)	82 (40.4)		742 (86.5)	116 (13.5)	
Length of stay*	-	-		4.51 (0.33)	3.04 (3.78)	-
Admission type						
Appropriate	-	-		852 (94.7)	48 (5.3)	<0.001
Inappropriate	-	-		460 (83.8)	89 (16.2)	

*average lengths of appropriate and inappropriate days per hospitalization (SD).

### Factors associated with inappropriateness

Results of univariate analyses are shown in Table 2. Residence–Shanghai (odds ratio [OR] = 0.380, 95% confidence interval [CI] = 0.233–0.618), admission route–outpatient (OR = 0.423, 95% CI = 0.187–0.959), readmission–yes (OR = 0.573, 95% CI = 0.350–0.939), and service type–surgical (OR = 4.002, 95% CI = 2.411–1.287) were significantly associated with inappropriateness of admissions. In addition, a higher prevalence of inappropriate hospital days was found in non-Shanghai (8.80 ± 21.54), self-pay (8.12 ± 21.35), readmitted (8.69 ± 20.44), service type–medical (8.87 ± 22.08), no comorbidity (7.19 ± 19.99), and inappropriate admission (0.344) patient groups. To more clearly illustrate how inappropriateness was distributed, median and IQR of subgroups after dropping 0 values are presented below. The median percentage of inappropriate hospital days and IQR were 19.05 (IQR = 37.63) and 33.33 (IQR = 41.67) for Shanghai and non-Shanghai residents, respectively; 50.00 (IQR = 35.00) and 13.81 (IQR = 11.53) for self-pay and insured patients, respectively; 39.22 (IQR = 41.90) and 20.87 (IQR = 33.68) for readmitted and non-readmitted children, respectively; 33.33 (IQR = 35.78) and 29.17 (IQR = 41.07) for surgical and medical service types, respectively; 20.00 (IQR = 15.91) and 35.42 (IQR = 42.93) for inpatients with comorbidity and without comorbidity, respectively; and 50.00 (IQR = 45.12) and 21.74 (IQR = 17.73) for the inappropriate admissions and hospital days, respectively.

**Table 2:** Univariate analysis results.

		Admissions			Hospital stay	
	OR	95% CI		*p*	Percentage of inappropriate days % (SD)	*p*
		Lower	Upper			
Gender						
Male	0.89	0.553	1.425	0.622	5.45 (17.23)	0.694
Female	-	-	-	-	6.26 (17.87)	
Age						
0.5 < and ≤ 5	0.875	0.537	1.425	0.592	2.38 (8.91)	0.147
5 < and ≤ 9.5	1.279	0.781	2.094	0.329	2.74 (11.22)	
9.5 < and ≤ 14	-	-	-		7.11 (19.45)	
Residence						
Shanghai	0.380	0.233	0.618	<0.001	3.00 (11.90)	0.005
Non-Shanghai	-	-	-	-	8.80 (21.54)	
Payment method						
Self-pay	1.202	0.736	1.964	0.463	8.12 (21.35)	0.004
Insured	-	-	-		1.99 (5.90)	
Day of admission						
Holiday	0.711	0.420	1.202	0.202	-	
Working day	-	-	-		-	
Admission time						
08:00–11:59	0.875	0.537	1.425	0.592	-	
12:00–17:59	1.279	0.781	2.094	0.329	-	
18:00–07:59	-	-	-	-	-	
Admission route						
Outpatient	0.423	0.187	0.959	0.039	5.71 (18.09)	0.708
Emergency	-	-	-		7.06 (9.79)	
Readmission						
Yes	0.573	0.350	0.939	0.027	8.69 (20.44)	0.022
No	-	-	-		3.90 (14.97)	
Service type						
Surgical	4.002	2.411	6.642	<0.001	2.81 (10.49)	0.003
Medical	-	-	-		8.87 (22.08)	
Comorbidity						
Yes	0.763	0.453	1.287	0.311	2.69 (8.86)	0.043
No	-	-	-		7.19 (19.99)	
Length of stay*	-	-	-	-	0.344	<0.001
Admission type						
Appropriate	-	-	-	-	1.54 (6.17)	<0.001
Inappropriate	-	-	-		12.69 (25.73)	

* Spearman’s rho correlation coefficient.

The results of multivariate analyses are presented in Tables 3 and 4 for admissions and hospital days, respectively. In the full model for inappropriate admissions (log-likelihood = 335.388, *χ*^2^ = 50.505, *p* < 0.001), patients living in Shanghai (OR = 0.327, 95% CI = 0.192–0.557, *p* < 0.001) and/or admitted through the outpatient sector (OR = 0.367, 95% CI = 0.148–0.908, *p* = 0.030) were found to be associated with a lower risk of being admitted inappropriately. Children patients admitted for surgical services were reported to have a higher risk of inappropriate admission (OR = 4.039, 95% CI = 2.382–6.850, *p* < 0.001). In the full model for prevalence of inappropriate hospital days (*R*^*2*^ = 0.228, adjusted *R*^*2*^ = 0.217, *p* < 0.001), self-pay (*β* = 5.349, 95% CI. = 1.535–9.163, *p* = 0.006), longer length of stay (*β* = 0.403, 95% CI. = 0.112–0.693, *p* = 0.007), and inappropriate admission (*β* = 13.557, 95% CI. = 9.623–17.490, *p* < 0.001) were determined to be associated with a greater percentage of inappropriate hospital days. Moreover, service type–surgery (*β* = -8.629, 95% CI. = -12.587 - -5.671, *p* < 0.001) and comorbidity–yes (*β* = -4.901, 95% CI. = -8.884 - -0.919, *p* = 0.016) were reported to be associated with a lower level of inappropriate hospital days.

**Table 3:** Multivariate logistic regression analysis of inappropriate admissions.

	*β* coefficient	OR	SE	95% CI		*p*
				Lower	Upper	
Residence–Shanghai	-1.118	0.327	0.272	0.192	0.557	<0.001
Admission route–outpatient	-1.003	0.367	0.463	0.148	0.908	0.030
Service type–surgical	1.396	4.039	0.270	2.382	6.850	<0.001

**Table 4:** Multivariate regression analysis of the percentage of inappropriate hospital days.

	*β* coefficient	95% CI *β* coefficient		SE	Standardized *β* coefficient	*p*
		Lower	Upper			
Payment method–self-pay	5.349	1.535	9.163	1.938	0.148	0.006
Service type–surgical	-8.629	-12.587	-4.671	2.011	-0.247	<0.001
Comorbidity–yes	-4.901	-8.884	-0.919	2.023	-0.129	0.016
Length of stay	0.403	0.112	0.693	0.147	0.151	0.007
Admission type–inappropriate	13.557	9.623	17.490	1.998	0.378	<0.001

### Reasons for inappropriateness

Reasons for inappropriate admissions and hospital days are listed in Table 5. Premature admission (n = 85, 75.9%) and awaiting test results (n = 47, 34.3%) were identified to be the most frequent reasons for inappropriate admissions and hospital days, respectively. Premature admission refers to that patients were admitted before any tests, surgery, and other services were arranged. Patients that needed a lower level of institutional care and care that can be administered on an outpatient basis constituted 9.8% (n = 11) and 8.9% (n = 10) of inappropriate admissions, respectively. Six patients (5.4%) were inappropriately admitted due to inconvenience of transportation. Specifically, all six of these children had difficulty with mobility and lived outside of Shanghai, and thus waiting outside of the hospital could be expensive. Awaiting surgery, test execution, test scheduling, discharge, and consultation resulted in 19.7% (n = 27), 10.9% (n = 15), 10.2% (n = 14), 8.8% (n = 12), and 5.1% (n = 7) of inappropriate days of care, respectively. 10.9% (n = 14) of the inappropriate days were attributable to non-preparation of family care, and the children had to wait in the hospital until home care became available.

**Table 5:** Reasons for inappropriate admissions and hospital days.

Reasons for inappropriateness	N	%
Inappropriate admissions		
Premature admission	85	75.9
Patient needs care at a lower level than a secondary hospital	11	9.8
Patient needs care that can be administered on an outpatient basis	10	8.9
Inconvenience of transportation	6	5.4
Inappropriate hospital days		
Awaiting test results	47	34.3
Awaiting surgery	27	19.7
Awaiting test execution	15	10.9
Family unprepared for home care	15	10.9
Awaiting test scheduling	14	10.2
Awaiting discharge	12	8.8
Awaiting consultation	7	5.1

## Discussion

Cost-effectiveness and efficiency are of pivotal importance for developing countries with limited health resources, and the current study presents the first empirical evidence of children’s hospital utilization appropriateness in China. Of the 291 admissions and 1449 hospital days reviewed, the overall inappropriateness rate of admissions and stays were 38.5% and 9.5%, respectively. Although the inappropriateness of hospital days is moderate compared to that found in international studies, the high prevalence of inappropriate admissions should be emphasized.

According to the multivariate regression analysis, patients who lived outside of Shanghai were found to have a higher risk of being admitted inappropriately. Residence was also reported to be associated with children’s hospital admission appropriateness in a study performed in Italy [11]. In our sample, approximately half of the inpatients were non-Shanghai residents. However, unlike the patients in the Italian study, who were mostly hospitalized through admission planning, the inpatients in our sample were self-referred. Since no compulsory gate-keeper exists in China, families intend to seek health service providers in Shanghai to obtain better perceived quality of care [24]. It is commonly agreed that the level of hospital appropriateness can serve as an approximate indicator of perceived accessibility and quality of primary care [13, 25]. To improve appropriateness by non-Shanghai residents, possible solutions could be through enhancing health resources planning and improving the quality of care nationally. In addition, considering that over half of the children (52.1%) who were without Shanghai residence were having surgical services in the hospital, and premature admissions and awaiting test results were the most important causes of inappropriateness, it could also be useful for improving appropriateness if pre-surgery tests can be arranged more efficiently.

Admissions through the emergency sector were identified to be associated with a higher risk of inappropriate admission. This result is unanticipated because, in most of the extant literature, concerning evaluations of hospital use by either adults or children, greater levels of inappropriateness were observed in admissions via outpatient sectors [11, 19, 26]. Potential explanations for this phenomenon include misuse of emergency resources by parents and poor communication between internal sectors in this hospital [26]. For instance, Morreson suggested that providing adequate health education to parents can greatly augment appropriate emergency department use of children [27]. Moreover, there is also a possibility that in emergency context, observation time is limited and pressures and tensions of the patient-doctor relationship are higher. However, further investigation is required to validate this assertion.

Research has also identified service type to be a common risk factor of inappropriateness. The results, however, are mixed. For example, research teams from Italy and Spain reported a higher rate of inappropriate admissions for medical hospitalizations [28, 29]. However, in our study, surgical service was associated with a higher prevalence of inappropriate admissions and lower rates of inappropriate length of stay. This is in accordance with the result that the most frequent reasons for inappropriateness were premature admission and awaiting test results, because surgical hospitalizations usually require a certain diagnosis, treatment, and test to be performed prior to surgery, and medical hospitalizations often necessitate the results of tests to decide further treatments.

Self-pay was determined to be associated with a higher prevalence of hospital days. This result is similar to the results of adult hospitalization screening studies in China [18]. In numerous developed countries and regions, care for children is free-of-charge. In Shanghai, one of the most developed cities in China, residents’ children’s inpatient costs are covered by “double insurances”: Urban Residents Health Insurance (URBHI) and Children’s Hospitalization Fund (CHI). URBHI covers the first half of inpatient costs, and CHI covers the remaining costs with a ceiling. To be insured by these two insurances, the parent of the child is required to have Shanghai residency. As part of the basic medical insurance, URBHI contains costs by listing reimburse-able treatments and a global budget contract with providers. CHI covers the other half of the inpatient costs, and it contains costs on the demand side by a ceiling of 200 thousand CNY for each insured person per year. Inpatient costs by insured children of non-Shanghai residents can be reimbursed by a certain percentage, according to different local policies. However, no such limit is imposed on self-pay patients. Longer length of stay and higher health expenses of self-pay patients were observed in several studies conducted in China. According to cost-shift and target income theories, self-pay patients are expected to have higher levels of inappropriateness.

Comorbidity and length of stay served as approximate indicators of complexity and severity of disease in our research. Patients with comorbidity are usually in relatively worse condition and need more intensive care, and thus lower rates of inappropriateness were reported. Interestingly, the association between comorbidity and percentage of inappropriate hospital days was not significant in the assessment of adults in Shanghai [18]. This difference might imply that parental or physician over-caution could play a key role in children’s inappropriate hospital utilization. For example, some prospective studies performed in regional hospitals indicated that parental anxiety can induce substantial inappropriate hospital use [8, 9]. However, this hypothesis needs further investigation into the entire hospitalization process with physicians and parents. Contrary to our hypothesis, a longer length of stay was associated with higher rates of inappropriateness, because shorter hospital days are considered to be of less complexity and variation in practice. This association confirms the assertion that length of stay is not an effective disease complexity/severity indicator [11], but rather a valid measure for service efficiency in this context.

Inappropriateness of admission is correlated with higher prevalence of inappropriate hospital days. This association is also widely observed in both pediatric and adult utilization review studies [11, 12, 30, 31]. According to the criteria of the C-PAEP, an inappropriate admission indicates that the patient either needed less intensive services or had less severe symptoms, and it is reasonable that the extent of inappropriate hospital days of these admissions would be higher.

Premature admission and awaiting test results constituted the main reasons for inappropriate admissions and hospital days, respectively. Specifically, three-fourths of the inappropriately admitted children were premature admissions. These patients needed hospital care, but all requisite services did not take place on the same day of admission. Premature admission is a common reason for inappropriateness in both children and adult hospital utilization review studies [20, 32, 33]. In our sample, the majority of the premature admissions were related to surgical stays (54.1%, n = 46). Test-related delays constituted 55.4% (n = 76) of inappropriate hospital days. Among these causes, awaiting test results comprised approximately one-third (n = 47, 34.3%) of the total inappropriateness. This justified our result that patients hospitalized in surgical wards had higher rates of inappropriateness. Former studies have demonstrated that admission protocol, for instance, performing pre-operative diagnosis and investigation on an outpatient basis for elective surgeries, could significantly reduce inappropriate days of care [7]. It is suggested that better admission planning and test arrangements could constitute effective measures to improve hospital utilization, especially for surgical stays. Additionally, from the demand-side perspective, educating the public to improve the patients’ awareness and healthcare service behavior is also of vital importance. There are several limitations of this study. First, our research was conducted in one secondary hospital in central Shanghai, the reliability and validity of the Chinese Version PAEP (C-PAEP) were tested in the same hospital of our study. Although records of all children patients admitted to the hospital from 1 June 2016 to 1 June 2019 were included, the sample size is relatively small compared to other multi-center studies, especially in 6 months- to 5 years-old group. Therefore, our sample is only representative of similar hospitals in Shanghai. We suggest that other hospitals should always perform a reliability review with their own hospital samples first before using the C-PAEP. Interested researchers could consider using the C-PAEP to evaluate utilization appropriateness of primary pediatric hospitals and tertiary hospitals. However, we strongly recommend researchers to adapt the C-PAEP to the specific context of practice prior to review. Second, even though we included as many factors as possible which were identified in the literature in our study, we may have missed some variables that could affect the findings. We hope that subsequent research can identify such additional factors to increase the robustness and generalizability of the results of this study. Besides, the decision of hospitalization of a child can be much more complex than that of an adult. Parental knowledge, experience, and other factors, for example, parental anxiety, can influence the decision-making of a pediatrician [9, 12], and this is the reason that our research did not differentiate internal and external inappropriateness in our analysis. However, the retrospective nature of this study makes it impossible to reflect on these critical determinants of inappropriateness. The inclusion of such societal factors can be useful for policy design and hospital management improvement. Future studies should consider concurrent or prospective use of the C-PAEP to include parental factors into appropriateness analysis. Third, only inappropriate utilization was analyzed in this research. A more comprehensive understanding of hospital utilization can be presented if the characteristics and distribution of appropriateness criteria were explored. Finally, like all retrospective studies, our results rely heavily on the quality of medical records. Although the results of inappropriateness are reliable, there might be some critical information missing for review, for instance, reasons for readmission, main symptoms for admissions with clear diagnoses, etc. Interested researchers should consider using the C-PAEP concurrently or prospectively to avoid this drawback resultant from insufficient information.

## Conclusions

This research presents the first hospital utilization review evidence of children in China’s context of practice, and provides comparable results of inappropriateness evaluation. Inappropriate admissions and hospital days constituted 38.5% and 9.5%, respectively. Analysis of the associated factors and causes of inappropriateness indicated that interventions could be applied to substantially improve children’s hospital utilization.

## Supporting information

S1 File Criteria of the C-PAEP. (DOCX)
