# Supplementary material for: Prevalence and associated factors of inappropriate hospital admissions and days of children in a secondary hospital in Shanghai, China
Source: PLoS One. 2022 Oct 6;17(10):e0275645. doi: 10.1371/journal.pone.0275645 (PMC9536573; doi:10.1371/journal.pone.0275645)
Supplement: S1 File — (DOCX) [file pone.0275645.s001.docx]

**Criteria of the C-PAEP**

| **Admission Part** |
| --- |
| 1. Severity of Illness Criteria |
| 1. Sudden onset of unconscientiousness (coma or unresponsiveness) or disorientation |
| 1. Acute or progressive sensory, motor, circulatory, or respiratory embarrassment sufficient to incapacitate the patient (inability to move feed, breath, urinate, etc.) |
| 1. Acute loss of sight or hearing |
| 1. Acute loss of ability to move body part |
| 1. Persistent fever ($\geq$37.8$℃$ orally or $\geq38.3℃$ rectally) for more than 5 days |
| 1. Active bleeding |
| 1. Wound dehiscence or evisceration |
| 1. Severe electrolyte/acid base abnormality (any of the following values): 2. Na $<$ 123 mmol/L,   Na $>$ 156 mmol/L   1. K $<$ 2.5 mmol/L,   K $>$ 5.6 mmol/L   1. CO_2_ combining power (unless chronically abnormal) $<$ 20 mmol/L,   CO_2_ combining power (unless chronically abnormal) $>$ 36 mmol/L   1. Arterial pH $<$ 7.30,   Arterial pH $>$ 7.45 |
| 1. Hematocrit $<$ 30% |
| 1. Pulse rate greater or less than the following ranges (optimally a sleeping pulse for $<$ 12 years old):   6 months to 2 years minus 1 day of age, 80-100/min;  2-6 years of age, 70-200/min;  7-11 years of age, 60-180/min;  $\geq$12 years of age, 50-140/min |
| 1. BP values outside following ranges:   6 months-2 years minus 1 day of age, 70-100/40-85 mm Hg;  2-6 years of age, 75-125/40-90 mm Hg;  7-11 years of age, 80-130/45-90 mm Hg;  $\geq$12 years of age, 90-200/60-120 mm Hg |
| 1. Need for lumbar puncture |
| 1. Any of the following conditions not responding to outpatient (including emergency room) management: 2. Seizures 3. Cardiac arrhythmia 4. Bronchial asthma or croup 5. Dehydration 6. Encopresis (for cleanout) 7. Severe sepsis 8. Acute abdomen 9. Other physiologic problem |
| 1. Special pediatric problems: 2. Child abuse 3. Noncompliance with necessary therapeutic regimen 4. Need for special observation or close monitoring of behavior, including calorie intake in cases of failure to thrive |
| 1. Intensity of Service |
| 1. Surgery or procedure scheduled within 24 hours necessitating 2. General or regional anesthesia or 3. Use of equipment, facilities, or procedure available only in a hospital |
| 1. Treatment in an intensive care unit |
| 1. Vital sign monitoring every 2 hours or more often (may include telemetry or bedside cardiac monitor) |
| 1. IV medications and/or fluid replacement (does not include tube feedings) |
| 1. Chemotherapeutic agents that require continuous observation for life-threatenning toxic reaction |
| 1. Intermittent or continuous respirator use at least every 8 hours |
| **Day of Care Part** |
| 1. Medical Services |
| 1. Procedure in operating room that day |
| 1. Procedure scheduled in operating room the next day, necessitating preoperative consultation or evaluation |
| 1. Cardiac catheterization that day |
| 1. Angiography that day |
| 1. Biopsy of internal organ that day |
| 1. Thoracentesis or paracentesis that day |
| 1. Invasive CNS diagnostic procedure that day (eg, lumbar puncture, cisternal tap, ventricular tap, pneumoencephalography) |
| 1. Gastrointestinal endoscopy that day |
| 1. Any test requiring strict dietary control for the duration of the diet |
| 1. New or experimental treatment requiring frequent dose adjustments with direct medical supervision |
| 1. Close medical monitoring by a doctor at least three times daily (observations must be documented in record) |
| 1. Postoperative day for any procedure described in numbers 1 or 3 to 8 above |
| 1. Nursing/Life Support Services |
| 1. Respiratory care – intermittent or continuous respirator use and/or inhalation therapy (with chest physical therapy, intermittent positive pressure breathing) at least three times daily, isoetharine hydrochloride (Bronkosol) with oxygen, oxyhoods, oxygen tents |
| 1. Parenteral therapy – intermittent or continuous IV fluid with any supplementation (electrolytes, protein, medications) |
| 1. Continuous vital sign monitoring, at least every 30 minutes for at least 4 hours |
| 1. Intake and/or output measurement |
| 1. Major surgical wound and drainage care |
| 1. Traction for fractures, dislocations, or congenital deformities |
| 1. Close medical monitoring by nurse at least three times daily with doctor’s orders |
| 1. Patient Conditions |
| (Being reviewed the day before the day of care)   1. Inability to void or move bowels, not attributable to neurologic disorder – usually a postoperative problem |
| (Being reviewed within 2 days before the day of care) |
| 1. Transfusion due to blood loss |
| 1. Ventricular fibrillation or ECG evidence of acute ischemia, as stated in progress note or in ECG report |
| 1. Fever at least 38.3$℃$ rectally (at least 37.8$℃$ orally), if patient was admitted for reason other than fever |
| 1. Coma – nonresponsiveness for at least 1 hour |
| 1. Acute confusional state, including withdrawal from drugs and alcohol |
| 1. Acute hematologic disorders – significant neutropenia, anemia, thrombocytopenia, leukocytosis, ertythrocytosis, or thrombocytosis – yielding signs or symptoms |
| 1. Progressive acute neurological difficulties |
